# Comparison of Two Different Accesses Single Orifice Percutaneous Endoscopic Surgery in Diagnosis of Ascites of Unknown Origin: A Retrospective Study

**DOI:** 10.1155/2022/1127400

**Published:** 2022-04-04

**Authors:** Li-liangzi Guo, Ben-hua Wu, Zhi-Chao Yu, Ming-han Luo, Feng Li, Feng Xiong, Cheng Wei, Rui-yue Shi, Ting-ting Liu, Ding-guo Zhang, De-feng Li, Zheng-lei Xu, Hui-ming Zhu, Li-sheng Wang, Jun Yao

**Affiliations:** ^1^Department of Gastroenterology, Shenzhen People's Hospital (the Second Clinical Medical College, Jinan University; The First Affiliated Hospital, Southern University of Science and Technology), Shenzhen, Guangdong, China; ^2^Department of Gastroenterology, The First Affiliated Hospital, Jinan University, Guangzhou, Guangdong, China; ^3^Department of Gastroenterology and Hepatology, Christus Trinity Mother Frances Hospital and Clinic, Tyler, TX, USA

## Abstract

**Background:**

Ascites is a common clinical finding caused by many different diseases, so we developed a technique termed single orifice percutaneous endoscopic surgery (SOPES) which can access peritoneal cavity through the contralateral McBurney's point or umbilicus to seek the underlying causes. In this study, we describe the initial clinical experience of SOPES and compare the application of two accesses.

**Methods:**

This is a retrospective study performed between 2007 and 2018. Patients with ascites of unknown origin who underwent these two kinds of SOPES were included. Main outcomes were measured by diagnostic accuracy, complication rate, procedure time, time till stitches removal, length of hospital stay, and hospital cost.

**Results:**

148 patients successfully undergone SOPES via the contralateral McBurney's point (IM group, *n* = 70) or the umbilicus (UM group, *n* = 78). 63 patients in the IM group and 71 patients in the UM group reached clear diagnosis (90.0% vs. 91.0%, *p* = 0.831). The overall complication rate was 5.4%, while the UM group was higher than the IM group (10.3% vs. 0%, *p* = 0.017). All complications were resolved after medical treatment, and no mortality resulted from this procedure. The procedure time and the time until stitches removal in the UM group were longer than that in the IM group. There were no significant differences in length of hospital stay and hospital cost between the two groups.

**Conclusions:**

SOPES, which combines the strength of minimally invasive single orifice incision and flexible angles of examination and instrumentation, is a newly developed flexible endoscopic surgical modality that provides new important clinical valuable in evaluation of ascites of unknown origin. Moreover, SOPES via the contralateral McBurney's point was safer than the umbilicus approach.

## 1. Introduction

Ascites is a common clinical finding that is caused by many different underlying diseases, such as cirrhosis, tuberculosis peritonitis, malignancies, congestive heart failure, nephrotic syndrome, and pancreatitis. Although the most frequent cause of ascites is liver cirrhosis [1], many other diseases also produce ascites. In most cases, the cause of ascites can be definitively diagnosed after comprehensive examinations such laboratory tests (ascitic fluid biochemistry, cytology, culture, and tumor markers) and imaging studies (X-ray, ultrasound, and CT/MRI). However, sometimes the cause cannot be definitely diagnosed without further investigation, which may include invasive approaches.

Invasive options include conventional laparoscopic surgery, single-incision laparoscopy surgery (SILS), and natural orifice transluminal endoscopic surgery (NOTES). Diagnostic laparoscopy plays an important part in the evaluation of ascites of unknown origin [2], but it requires a rigid endoscopy. Although the initial trials with NOTES were performed via a transgastric route, other routes such as transvaginal [3], transcolon [4], transanal [5], and transumbilical [6] were subsequently reported. The flexibility of NOTES for careful observation is recommendable. However, major barriers that limit the clinical application of NOTES include difficulty with access, closure, infection, devices, and spatial orientation [7].

Inspired by these studies, we developed another technique, known as single orifice percutaneous endoscopic surgery (SOPES), a procedure that combines benefits of both laparoscopic surgery and NOTES. In this study, we compared two different accesses of SOPES, which were located at the contralateral McBurney's point and the umbilicus, and then applied SOPES in the diagnosis of ascites of unknown origin. The aim of our study was to describe the initial clinical experience of SOPES and to compare the pros and cons of these two kinds of SOPES.

## 2. Methods

### 2.1. Study Design

This was a retrospective study. The study protocol was approved by the Institute Research Ethics Committee of Shenzhen People's Hospital and the Second Clinical Medical College of Jinan University. All patients had a signed informed consent agreement before the procedure.

Patients were eligible for SOPES when they met the following inclusion criteria: (1) Ascites was confirmed by physical examination and/or ultrasound. (2) Blood examination, including inflammatory markers, tumor markers, bacterial cultural, PPD test, and more, did not yield a clear diagnosis. (3) The examination of the ascites fluid, including biochemical tests, cytology, culture, and tumor marker, did not reveal a clear diagnosis. (4) A definitive diagnosis was not concluded by conventional radiological imaging, involving an abdominal radiograph, CT/MRI and other techniques. (5) Routine endoscopy (gastroscopy and colonoscopy) and/or tissue sampling did not allow a positive diagnosis. (6) Conservative treatment was ineffective.

Patients with contraindications to this procedure were excluded as follows: (1) severe cardiopulmonary disorder, respiratory or renal failure, and sepsis; (2) pregnancy or menstrual period; (3) the history of recent abdominal and/or pelvic surgery; (4) administration of anticoagulant or nonsteroidal anti-inflammatory drug therapy in the preceding week; and (5) coagulation disorders.

### 2.2. Instruments

The endoscope (GIF-XQ260, Olympus Corp, Tokyo, Japan) was sterilized with ethylene oxide gas for 3 hours at 55°C and then aeration cycle for 12 hours. Two air sterilizers (KJF600, Sturdy, Shenzhen, China) were switched on for 2 hours before SOPES. The multifunction negative pressure flow cleaning machine was in operation during the percutaneous endoscopic surgery. The microbial contamination of the atmosphere in the endoscopy room was detected every month using the plate exposure method. The bacterial count of the plate was less than 4.0 CFU (colony-forming unit), which remains within hygiene standards for disinfection in hospitals.

### 2.3. SOPES Procedure

#### 2.3.1. Regular Preparation

**Figure 1:**
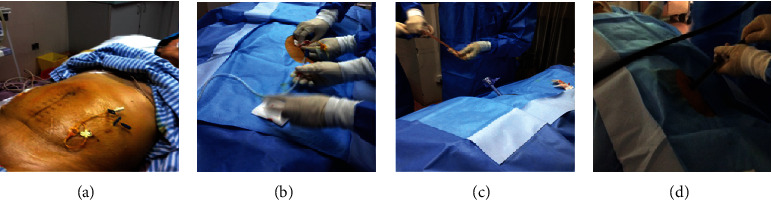
The main procedure of SOPES via the contralateral McBurney's point. (a) An double-lumen central venous catheter was placed in advance. (b) Insufflation through the catheter to establish pneumoperitoneum. (c) A 10-mm trocar cannula was inserted in the dilated access. (d) The endoscope was passed through the trocar to scrutinize peritoneal cavity.

All patients were deprived of food and water for at least 8 hours before the start of procedure. In the IM group, patients had undergone an abdominal paracentesis with ultrasound guidance the day before SOPES, leaving a double-lumen central venous catheter in the abdomen (Figure 1(a)). However, patients in the UM group did not need this step. After preoperative preparation, the procedure was carried out under deep sedation anesthesia with pethidine 50 mg and midazolam 5 mg. The patient was supine with electrocardiogram monitoring. Carbon dioxide insufflation was used during the procedure, and continuous monitoring of blood pressure, pulse, oxygen saturation, abdominal pressure, and airway pressure was performed. The operator adhered to strict aseptic principles. All SOPES were conducted by two skilled endoscopists (Li-sheng Wang and Hui-ming Zhu) who had performed about 200 cases of NOTES and more than 500 cases of ESD for lesions of the upper and lower digestive tract.

#### 2.3.2. Surgical Approach and Artificial Pneumoperitoneum

*(1) SOPES via the Contralateral McBurney's Point*. The contralateral (left) McBurney's point is located one-third of the distance between the left anterior superior iliac spine (ASIS) and the umbilicus, which is the favored anatomical landmark for paracentesis. The double-lumen central venous catheter at the contralateral McBurney's point can be used as a probe. The first step was to insufflate through the catheter to establish pneumoperitoneum (Figure 1(b)). An abdominal pressure between 11 and 13 mmHg was continuously monitored by the pneumoperitoneum machine. Insufflation was stopped when the pressure exceeded 15 mmHg. While the pneumoperitoneum was established, the central venous catheter was replaced with a rigid guide wire by means of wire catheter exchange technique. The endoscopist made a 1-cm skin incision across the small hole of central venous catheter.

**Figure 2:**
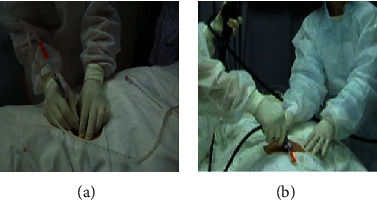
The main procedure of SOPES via the umbilicus. (a) The pheumoperitoneum was established through the umbilicus using Veress needle. (b) The endoscope was passed through the trocar to overview the peritoneal cavity.

*(2) SOPES via the Umbilicus*. Without the double-lumen central venous catheter in abdomen, the endoscopists made a 1-cm skin incision which was about 0.5 cm distance from the umbilicus (Figure 2(a)). Compared to the contralateral McBurney's point approach, the pneumoperitoneum was directly established using a Veress needle. The abdominal pressure was controlled in the same way as the IM group.

#### 2.3.3. Inspection Method

**Figure 3:**
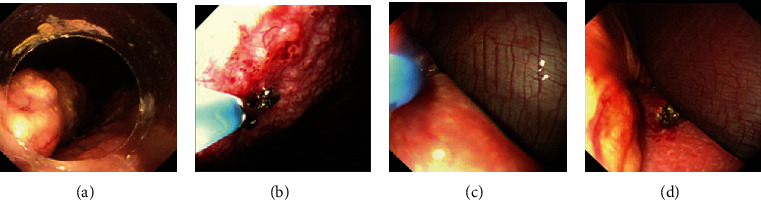
The visualization and manipulation under SOPES. (a) Observation of the peritoneal cavity. (b) Biopsy under direct vision. (c, d) APC was used in case of bleeding.

Under the guidance of a rigid wire, dilatation of the access port was performed bluntly by a cone shape expansion bougie (5 mm, 7 mm, 9 mm, 11 mm, and 12.8 mm diameter in sequence). We inserted a 10-mm trocar cannula in the dilated access port, which was similar to that used in laparoscopic surgery (Figure 1(c)). Then, the flexible endoscope passed through the trocar (Figure 1(d) and Figure 2(b)) to examine the peritoneal cavity, exploring anticlockwise from the right lower quadrant of abdomen to the right upper quadrant (Figure 3(a)), making sure that the abdomen was examined in a systematic and comprehensive manner. Biopsies were performed using instruments through the endoscopic work channels at 3 or more sites that appeared abnormal (Figure 3(b)). Argon plasma coagulation (APC) was used in case of bleeding (Figures 3(c) and 3(d)). At the end of this procedure, it was necessary to ensure that there were no bleeding and perforation and to remove the gas from peritoneal cavity. The endoscopic tools were withdrawn through trocar. A drainage tube was placed into the peritoneal cavity and affixed to the abdomen with suture. Finally, the area of abdomen was disinfected and dressed.

#### 2.3.4. Definitions of Bleeding

Intraoperative bleeding was defined as oozing or pulsatile bleeding that necessitated the use of hemostatic forceps or APC during the procedure. Delayed bleeding was defined as one of the following: hematemesis, melena, or decrease in hemoglobin level of >2 g/dL after SOPES.

### 2.4. Postoperative Management

After completion of the procedure, patients were given flumazenil and naloxone. Patients were kept NPO for 24 hours and were given parenteral nutrition (PN) after the procedure. The prophylactic antibiotics (the third generation of cephalosporin) was administrated intravenously 30 min before the procedure. The duration of antibiotic therapy was adjusted according to different clinical manifestations. Blood tests obtained before the procedure were reexamined the first day after the procedure. Real-time monitoring of the electrocardiograph and vital signs was performed continuously during and 2 hours after the procedure.

### 2.5. Outcome Measurements

The main outcomes included procedure time, diagnostic accuracy, complication rate, time till stitches removal, length of hospital stay, and hospital cost. The clinical characteristics of the enrolled cases were collected as follows: age, sex, complaints, prior examinations, treatments, and effects. Biopsy specimens were obtained for pathologic analysis and/or immunohistochemical staining in order to analyze the accuracy of the diagnosis. Adverse events during the procedure included the rupture of solid organs, perforate of hollow organs, intraoperative bleeding, subcutaneous emphysema, pneumothorax, and a conversion to a surgical or laparoscopic procedure. Adverse events after the procedure included abdominal pain, abdominal distension, transient fever, peritonitis, incision infection, delayed bleeding, subcutaneous emphysema, bowel obstruction, and others.

### 2.6. Statistical Analysis

The sample size was 148. Categorical variables were examined as percentages. Continuous variables were represented as means ± standard (SD) or median and interquartile range (IQR) and were compared by using the student t-test or Mann–Whitney test. Pearson's chi-squared test or Fisher's exact test was used to analyze the categorical data. Data analysis was conducted using SPSS version 23.0 software package (SPSS Company Chicago, IL, USA) for Windows. *P* value<0.05 was considered indicative of statistical significance.

## 3. Results

### 3.1. Population

Over the past 11 years from January 2007 to December 2018, there were 5828 patients with ascites registered in Shenzhen People's Hospital. A total of 148 patients (56 males and 92 females, mean age 47.80 years, range 12-88 years) underwent the SOPES, either via the contralateral McBurney's point (*n* = 70) or the umbilicus (*n* = 78) for ascites of unknown origin.

### 3.2. Diagnostic Value

**Figure 4:**
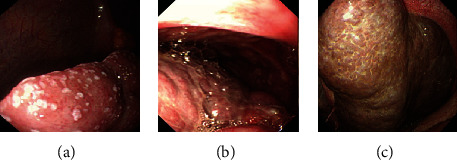
The pathologic changes of the liver. (a) Tuberculous peritonitis. (b) Hepatic carcinoma. (c) Liver cirrhosis caused by hemochromatosis.

All 148 patients tolerated SOPES successfully. Endoscopic biopsies were performed in 142 patients, and the total diagnostic rate was over 90.5%. 6 patients did not get biopsy specimen because of extensively dense adhesions (2 cases) or negative findings (4 cases). There were 24 cases of liver biopsy in total. The macroscopic condition of liver is shown in Figure 4. Hot biopsy forceps were used in case of need.

**Figure 5:**
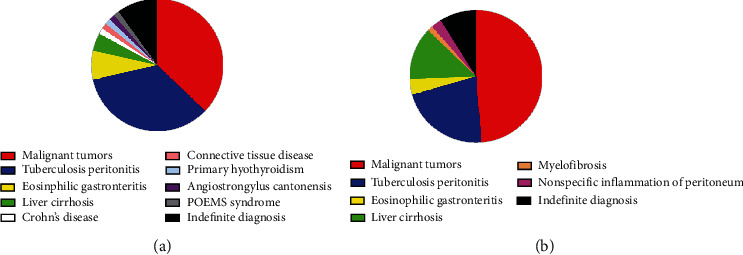
Final diagnosis of SOPES in patients of ascites of unknown origin. (a) IM group. (b) UM group.

According to the results of biopsy, endoscopic characteristics, and previous examinations, 63 patients (90.0%) in the IM group and 71 patients (91.0%) in the UM group were clearly diagnosed (*χ*2 = 0.045, *p* = 0.831). The total diagnostic value of these two kinds of SOPES is summarized in Figure 5.

The final diagnoses of the IM group included malignant tumors (26 cases), tuberculosis peritonitis (24 cases), eosinophilic gastroenteritis (5 cases), liver cirrhosis (3 cases), Crohn's disease (1 case), connective tissue disease (1 case), primary hypothyroidism (1 case), angiostrongylus cantonensis (1 case), and POEMS syndrome (1 case). In the 26 patients with peritoneal malignant tumors, malignant mesothelioma of peritoneum accounted for 7.69% (2 patients) and peritoneal metastatic cancers for 92.30% (24 patients). For patients with peritoneal metastatic cancers, oophoroma was diagnosed in 10 cases, gastric cancer in 1 case, hepatic carcinoma in 1 case, kidney cancer in 1 case, pancreatic cancer in 1 case, and extraosseous Ewing's sarcoma in 1 case. The remaining 7 cases did not yield a clear tumor origin.

The final diagnoses of the UM group included malignant tumors (38 cases), tuberculosis peritonitis (17 cases), liver cirrhosis (10 cases), eosinophilic gastroenteritis (3 cases), myelofibrosis (1 case), and nonspecific inflammation of peritoneum (2 cases). In the 38 patients with peritoneal malignant tumors, malignant mesothelioma of peritoneum accounted for 7.89% (3 patients) and peritoneal metastatic cancers for 92.11% (35 patients). For patients with peritoneal metastatic cancers, oophoroma was diagnosed in 8 cases, hepatic carcinoma in 7 cases, gastric cancer in 6 cases, colorectal cancer in 1 case, pancreatic cancer in 1 case, duodenal cancer in 1 case, and nasopharyngeal cancer in 1 case.

### 3.3. Adverse Events

SOPES was performed successfully in all 148 patients and 131 cases with no adverse events. The overall complication rate was 5.4%, 0% in IM group and 10.3% in UM group, respectively (*χ*2 = 5.72, *p* = 0.017). None of the patients required open laparotomy and/or conventional laparoscopic surgery during SOPES.

In the IM group, there were no severe adverse events during or after the procedure. However, some patients had transient and minor adverse events after the procedure such as fever (2 cases), slight distension (1 case), and abdominal pain (1 case).

In the UM group, complication during procedure occurred in one patient for widespread subcutaneous emphysema and pneumothorax caused by biopsy. So, the procedure was completed ahead of schedule. Among those who had successfully completed this procedure on time, 7 patients had varying degrees of postprocedure complications including puncture site infection (3 cases), delayed bleeding (3 cases), and mild subcutaneous emphysema (1 case). Among the 3 cases of delayed bleeding, 2 patients received exploratory laparotomy. One of them had an abdominal wall wound, and the bleeding was stopped by transfixion hemostasis. The other one had a liver puncture wound and then underwent the ligation of umbilical vein and a hemostasis of liver. Another one who did not convert to exploratory laparotomy received a CT scan demonstrating a well-defined soft tissue mass in the left rectus sheath, which was consistent with a rectus sheath hematoma. All of these complications were treated successfully, and no patients died due to this procedure. For the rest of patients who had minor adverse events after the procedure, 3 patients had a transient fever, and 2 patients had a slight abdominal pain.

### 3.4. A Comparison of Perioperative Outcomes

The procedure time was recorded from the beginning of the skin incision to the completion of the skin suture. The procedure time and the time until stitches removal in the IM group were shorter than in the UM group (*p* < 0.05). When comparing the length of hospital stay and hospital cost, there were no significant statistical differences between the two groups (*p* > 0.05). The data is summarized in Table 1.

## 4. Discussion

It is well-known that peritoneal biopsy is the gold standard approach for diagnosis, so the invasive diagnostic approach combined with a biopsy remains necessary. Diagnostic laparoscopy is a minimally invasive technique, which has long played an important part in the evaluation of ascites when the cause cannot be identified. Inspired by this dogma, we developed a new technique, termed “SOPES”, and applied it to the diagnosis of ascites of unknown origin.

In our study, patients with unexplained ascites received SOPES, and 134 patients were clearly diagnosed for an overall diagnostic rate of over 90.5%. It is shown that this is a novel, safe, and effective endoscopic method for difficult to diagnose ascites. Though the diagnostic rate of IM group was a little bit higher than the UM group, there was no statistical significance. The reason of this slight difference between two groups may probably be that the endoscope has a better view and flexibility in the abdominal cavity at the entrance of the contralateral McBurney's point. On the other hand, the paracentesis performed before SOPES allowed better visualization during this procedure.

**Figure 6:**
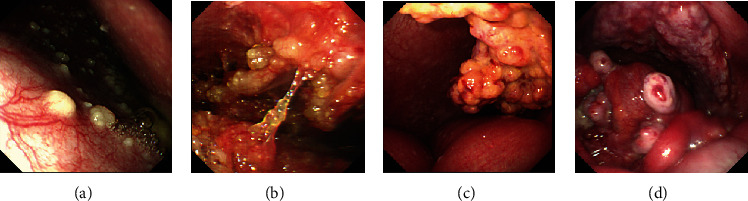
Appearance of malignant tumor. (a) Pathologic condition of irregular nodules on the parietal peritoneum and visceral. (b) Numerous jelly-like nodules on the parietal peritoneum and viscera. (c) An abdominal mass was found in the peritoneal cavity. (d) The wrapping and distorting of the greater omentum.

Chu et al. used laparoscopy to make a diagnostic evaluation of unknown origin ascites, finding that 60.5% were carcinomatosis peritonei, 20.2% were tuberculous peritonitis, 5.4% were cirrhosis, and 14.0% had no gross abnormality [2]. In our study, based on the final diagnoses, most patients had malignant tumors (43.2%) and tuberculosis peritonitis (27.7%); some had other diseases including liver cirrhosis (8.8%), eosinophilic gastroenteritis (5.4%), and others (5.4%); and a few received an indefinite diagnosis (9.5%). In the IM group, of the 26 patients with peritoneal malignant tumors, peritoneum mesothelioma accounted for 7.69%, and peritoneal metastatic cancers accounted for 92.30%. In the UM group, of the 38 patients with peritoneal malignant tumors, peritoneum mesothelioma accounted for 7.89%, and peritoneal metastatic cancers accounted for 92.11%. Malignant tumors with ascites as the first case also account for a large proportion of patients and are often difficult to detect by other imaging methods. 64 cases were diagnosed as peritoneal malignant tumors, including 5 peritoneum mesothelioma and 59 peritoneal metastatic cancers. Malignant mesothelioma of peritoneum is usually difficult to diagnose in our clinic. However, 5 cases were diagnosed as peritoneum mesothelioma by SOPES. The diagnostic SOPES combined with biopsy can help differentiate malignant disease from benign disease and trace the source of the malignant tumor (Figure 6). Pseudomyxoma peritoneum (PMP) is also a rare malignant disease which is always misdiagnosed as appendicitis or ovarian cancer. PMP always presents with altered bowel habits and infertility in women. It is characterized by a large volume of mucinous ascites and diagnosed by histology. In our study, a 44-year-old man, without clinical manifestations such as abdominal pain or distention, has ascites for 2 months and received a diagnostic SOPES that revealed numerous jelly-like nodules on the parietal peritoneum and viscera (Figure 6(b)). The 59 peritoneal metastatic cancers were derived from the oophoron (30.5%), stomach (15.3%), liver (13.6%), pancreas (5.1%), colon (6.8%), kidney (1.7%), duodenum (1.7%), nasopharynx (1.7%), extraosseous Ewing's sarcoma (1.7%), and unknown origins (22.0%). It follows that oophoroma is the most common peritoneal metastatic cancer. These results are consistent with previous study [8]. For those with no gross abnormality observed, this procedure was performed to exclude other diseases which may cause ascites. Thus, SOPES plays an important role in the diagnosis of ascites.

**Figure 7:**
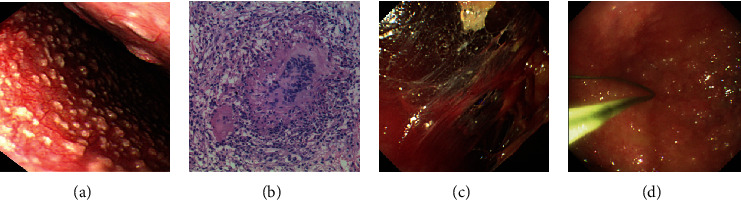
Appearances of tuberculosis peritonitis. (a) The parietal peritoneum and visceral were covered with widespread and numerous white miliary nodules. (b) Histology with hematoxylin-eosin staining revealed caseous necrosis (×10) in biopsied peritoneal lesions. (c) Severe adhesions between the peritoneum and intraperitoneal organ. (d) Caseous materials were found covering the abdominal wall and surface of the intraperitoneal organs.

Tuberculosis peritonitis should not be ignored in ascites of unknown origin because we found very large proportion of total patients with this disease. 6 patients did not receive a biopsy because of massive intra-abdominal adhesions or negative findings. The two patients who did not receive biopsy because of dense adhesion were recovered after treatment of antituberculosis drugs. Tuberculosis peritonitis (TBP), which affects an estimated 9.4 million cases globally in 2009, usually presents as fever, abdominal pain, distension, and ascites, but without characteristic or specific clinical features. It may go unrecognized and undiagnosed for months or even for years. The diagnostic SOPES could reduce the mortality rate due to delayed diagnosis. The efficacy of diagnostic laparoscopy [9] and NOTES [10] in TBP also has been well-acknowledged in current practice. Bhargava et al. described the macroscopic characteristic appearance of TBP by laparoscopy as thickened, hyperemic peritoneum with ascites; scattered, yellowish or whitish, granular, peritoneal nodules; and intra-abdominal fibroadhesive tissues [11]. These observations are identical to those found in our study (Figure 7).

At the same time, we could also find some relatively uncommon diseases by SOPES, such as eosinophilic gastroenteritis, myelofibrosis, and nonspecific inflammation of the peritoneum. Serositis is commonly seen in patients with connective tissue disease (CTD) such as systemic lupus erythematosus (SLE), where there is no macroscopic lesion in the peritoneal cavity. Lei Zhou et al. reported a patient who had massive and painful ascites as a prominent manifestation of SLE, in the absence of other well-recognized clinical features of SLE and the common causes of ascites [12]. Crohn's disease is a granulomatous inflammatory bowel disease which presents differently according to the lesion location, type, and complications. A case finally diagnosed as Crohn's disease initially presented with abdominal pain, fever, and weight loss. At first, it was challenging to differentiate it from intestinal tuberculosis and lymphoma because they have very similar clinical characteristics. The SOPES provides histological examination to reach a clear diagnosis.

The possibility of adverse events during and after SOPES is the most important concern. We performed SOPES successfully in all 148 patients and 131 cases with no serious adverse events. Subcutaneous emphysema and pneumothorax occurred in one patient during SOPES via the umbilicus. Pneumothorax is a known but rare complication of laparoscopic abdominal surgery, and it is most commonly seen in fundoplication [13]. The cause of pneumothorax might be the small preexisting defect in the diaphragm, which often occurs at the pleuroperitoneal hiatus, foramen of Bochdalek, the outer crus, or the esophageal hiatus [14]. The other postprocedure complications which were commonly reported include puncture site infection, bleeding, and mild subcutaneous emphysema. All the complications were relieved after symptomatic treatment. Compared with the UM group, patients in the IM group had no severe adverse events during or after procedure. The double-lumen central venous catheter, which was used to substitute the sharp pneumoperitoneum needle, might contribute to this outcome. The results suggest that although the two different accesses of SOPES contribute equally to the diagnostic value, SOPES via contralateral McBurney's point may bring patients less injury and better recovery than SOPES via umbilicus because of lower complication rate.

With the rapid development of endoscopy technology in recent years, we searched for SOPES as a newer means of access to the peritoneal cavity for less traumatic surgery and greater patient comfort. First, taking biopsy under direct vision and position could increase the diagnostic accuracy and prevent the occurrence of complications. Liver biopsy can be performed under direct vision through endoscopy SOPES. The advantage of SOPES is the same as conventional laparoscopy and SILS. It also indicates that SOPES could provide more space and effects as seen in other endoscopic surgeries. For example, the endoscopic approach of ESD or EMR could be used for the removal of small lesions in the abdominal cavity or on the surface of organs. Second, SOPES as a flexible and soft endoscopy can freely reach deeper parts of the abdominal cavity to ensure a complete observation and would not invert the position of abdominal organ at the same time. The SOPES combined the aesthetics of single-port approaches with the flexibility and safety of endoscopy. However, the standard laparoscopy requires 3 incisions on the abdomen, thus increasing the risk of bleeding and loss of aesthetic appearance of the abdominal wall. The intestine is fixed and lifted by the trocar, which is inserted through one of the incisions on the abdomen, in order to have a better visualization during laparoscopy. This behavior may potentially cause abdominal adhesion and adhesive intestinal obstruction in the long term. The SILS with a rigid endoscope is technically more difficult than standard laparoscopy [15], where all the instruments are packed together closely to limit the range of motion [16]. Third, the umbilicus can be developed as natural port for performing various procedures. This procedure can be satisfied with some people with special requirement of beauty and abdominal scars. In this respect, the procedure is similar with natural orifice transluminal endoscopic surgery (NOTES), which, many endoscopic physicians are showing great interest in, is a scar-less surgical procedure after laparoscopy. Comparing with the laparoscopic surgery, it may bring less pain, faster recovery, and better cosmesis. NOTES can be performed via transgastric, transvaginal, transcolonic, transanal, and other approaches. The major barriers that limit the clinical application of NOTES included access, closure, infection, devices, and spatial orientation [7]. Zhu JF et al. tried another access technique, trans-umbilical endoscopic surgery (TUES), which was based on the principle of NOTES, suggesting that TUES is easier technically when compared with NOTES [6]. Fourth, closure of the only incision on the abdomen is technically easier than laparoscopy and NOTES. We made only a 1-cm incision, while SILS necessitates a larger incision at the umbilicus, usually about 2-3 cm in diameter. It may bring less pain, shorter hospital stays, fewer finical burdens, and more rapid recovery. Therefore, SOPES combines the advantages of the above surgical methods and avoids the disadvantages of several operations to a certain extent.

## 5. Conclusions

The results of this study suggest that both contralateral McBurney's point and umbilicus are feasible approaches when performing SOPES in evaluation of ascites of unknown origin. SOPES via the contralateral McBurney's point seems to be safer with a lower complication rate when compared with SOPES via the umbilicus. Anyway, SOPES is a newly developed flexible endoscopic surgical modality which combines the strength of minimally invasive single orifice incision and flexible angles of examination and instrumentation. More prospective studies as well as clinical trials are required to further assess the application of SOPES in the future.

## Figures and Tables

**Table 1:** Clinical outcomes of IM group versus UM group for diagnosis of ascites of unknown origin.

Patients (*n* = 148)	IM group (*n* = 70)	UM group (*n* = 78)	*p* value
Gender, male/female	22/48	34/44	0.128
Age, (mean, range), years	46.1 (14-83)	49.2 (12-88)	0.276
Diagnostic value (*n*, %)	63/70 (90.0%)	71/78 (91.0%)	0.831
Malignant tumors	26 (41.3%)	38 (53.5%)	0.156
Tuberculosis peritonitis	24 (38.1%)	17 (23.9%)	0.076
Liver cirrhosis	3 (4.8%)	10 (14.1%)	0.069
Eosinophilic gastroenteritis	5 (7.9%)	3 (4.2%)	0.365
Others	5 (7.9%)	3 (4.2%)	0.365
Procedure time, (mean ± SD), min	38.16 ± 13.48	53.83 ± 13.69	≤0.001
Time till stitches removal (median, IQR), days	7 (7-7)	7 (7-7)	0.005
Complication rate (n, %)	0/70 (0%)	8/78 (10.3%)	0.017
During procedure	0 (0%)	1/8	
Pneumothorax	—	1	
Postprocedure	0 (0%)	7/8	
Delayed bleeding	—	3	
Infection of puncture site	—	3	
Subcutaneous emphysema	—	1	
Length of hospital stay, (median, IQR), days	2.59 (2.00-3.01)	2.92 (1.97-4.00)	0.222
Hospital cost, (mean ± SD), yuan	5940.57 ± 6301.21	6817.62 ± 10447.13	0.473

Abbreviations: IQR = interquartile range; SD = standard deviation.

## Data Availability

No data were used to support this study.
